# Wild Meets Domestic in the Near Eastern Neolithic

**DOI:** 10.3390/ani12182335

**Published:** 2022-09-08

**Authors:** Nerissa Russell

**Affiliations:** Department of Anthropology, Cornell University, Ithaca, NY 14853, USA; nr29@cornell.edu

**Keywords:** zooarchaeology, domestication, human–animal relations

## Abstract

**Simple Summary:**

Many recent theorists argue that reliance on binary oppositions to structure thought is a feature of the modern West, and therefore an inappropriate model for prehistory. While the world is usually more complicated than dualistic models, I suggest their use is not limited to modern Western cultures. Rather, we should determine whether they apply in each instance, and if the terms are defined in the same way. Here, I examine whether inhabitants of the earlier Neolithic in the Near East, the time of the first livestock herding, distinguished between wild and domestic animals. Lacking written records, I analyze imagery and animal remains. Zooarchaeologists use demographic profiles, size change, and other data to assess whether ancient people hunted or herded animals. I add contextual analysis to identify special treatment of animal remains that indicates they have ritual power, and choices in animal depictions. Three case studies each present windows into how animals were valued, showing that wild and domestic animals were treated differently, and thus a wild/domestic conceptual distinction existed. Moreover, differences between wild and domestic animals, and regional differences in the roles of specific species, shaped how both wild and domestic animals spread in the early days of agriculture.

**Abstract:**

The categories of wild and domestic are one of the classic ways the nature/culture dichotomy manifests itself in human interactions with the environment. Some argue that this distinction is not helpful and a projection of modern thought, and certainly the boundaries are complicated. However, we should try to determine in each case whether it was meaningful to particular people in the past. Here I explore whether wild and domestic were relevant concepts to the inhabitants of the Neolithic Near East in their relations with animals around the time when livestock herding began. Drawing on depictions of animals and the treatment of living animals and their remains, I examine three case studies (Cyprus, Upper Mesopotamia, and Çatalhöyük in central Anatolia) to evaluate whether emic distinctions between wild and domestic existed. I conclude that this was in fact a crucial distinction that shaped economic choices as well as ritual activities. Differential treatment of wild and domestic animals indicates that they were accorded different forms of personhood. The particular nature of human relations with wild animals helped shape the spread of both wild and domestic animals.

## 1. Introduction

Recent work grounded in relational or posthumanist theory rejects Cartesian dualisms such as nature/culture or wild/domestic, and such binaries are often cast as a product of modern Western thought [1,2,3,4,5,6]. There is much to be gained from such approaches; however, the tendency to reject all binary categories as hindrances to understanding and products of modern Western thought or a naturalistic ontology [7] itself creates a dualism between the West and the rest that is no less problematic.

Here I will explore a particular dualism: wild/domestic. In our relationships with other organisms, the nature/culture binary plays out principally through domestication. Rather than assuming either that wild and domestic are universal categories for farmers, or that only modern Western societies make this binary distinction, we should explore whether particular farming cultures distinguish conceptually between wild and domestic. Here I will focus on animals around the time of livestock domestication, drawing on the evidence of material remains of human–animal relations analyzed with the methods of archaeology and zooarchaeology (the study of animal remains from archaeological sites). I will argue that inhabitants of the Neolithic Near East did make this wild/domestic distinction, and indeed it is very likely that most people who keep domestic animals do so. However, these categories may not have the same meanings and implications in different societies and circumstances. Moreover, in all cases there are some human–animal relations that do not fall neatly into either category [8].

## 2. Domestication

Animal domestication is difficult to define with precision, and there is a long-running debate within and beyond zooarchaeology over how best to define it [8,9,10,11,12,13,14,15,16]. For this purpose, I will treat domestication as a relationship between humans and animals that spans generations (not just the taming of individual animals) and where animals belong to specific humans. I will focus on animals raised to serve human needs, rather than companion animals.

What we might loosely call herding has many effects, including bringing animals into the human sphere, engendering sustained relations between individual animals and humans, creating a new kind of property, and changing the conditions of animal lives. Husbandry practices vary, but always involve some degree of human control over animal movement and reproduction, and the provision of protection and care [9,17,18,19,20]. Herding alters the selective forces acting on animals in ways that change their bodies and their behavior [21,22,23,24,25].

Hunters tend to view wild animals as their equals, often as persons with souls, e.g., [26,27,28,29,30,31,32,33]. Domestic animals, although they may be objects of pride and affection, are usually seen as lesser beings, e.g., [34,35,36]. Moreover, while foragers engage in mutual relations of care with wild animals, often feeling that the natural world and its representatives such as animal spirits or spirit masters care for humans who behave properly, care flows primarily from humans to domestic animals [18,34,35,37].

Ethnographically, the wild/domestic distinction has been important to some non-Western societies, e.g., [38,39]. For instance, many New Guinea groups practice various forms of loose pig husbandry such that domestic pigs interbreed with wild (actually feral) ones and may spend much of their time roaming the bush on their own. Nevertheless, these groups make a strong conceptual distinction between domestic pigs owned by people and wild ones—even when the ‘domestic’ pigs were captured as infants from wild sows and raised in the village. One or the other may be required for specific ceremonies, and different rules apply [40].

## 3. Near Eastern Neolithic

The Near Eastern Neolithic has the earliest evidence of livestock domestication in the world, and animal domestication has been studied extensively there [41]. This focused research provides the resolution to address the nature of human–animal relations in early herding societies in this region. I am examining here the key regions in the transition to agriculture: the Fertile Crescent (Zagros, middle and upper Tigris and Euphrates basins [Upper Mesopotamia], and the Levant), central Anatolia, and Cyprus during the Pre-Pottery and Pottery Neolithic. There is, of course, variation across this area as well as within regions in the nature and timing of this process. Crudely, the Pre-Pottery Neolithic A (PPNA, ca. 9500–8500 calBC) is a time when plant cultivation was practiced widely in the region. Evidence of livestock herding appears in the PPNB (ca. 8500–6500 calBC). This is Levantine terminology and timing that I will extend to the rest of the region, although the adoption of pottery, and therefore the start of the Pottery Neolithic (PN) is earlier in some other parts of the Near East.

I will not discuss the process of animal domestication here, but rather focus on attitudes toward wild and domestic animals as it developed and spread. Suffice it to say that there is evidence for herding sheep by 8500 calBC in the Upper Mesopotamia region of southeast Turkey and northern Syria [42,43,44]; perhaps also goats around the same time in this area [43,44] but certainly by 8000 calBC in the Zagros foothills [45]; cattle perhaps as early as 8500 calBC in Upper Mesopotamia [46] but clearly shortly after 8000 calBC [47]; and pigs by ca. 7500 calBC in Upper Mesopotamia but with a gradual process beginning several centuries earlier [44,48,49,50].

## 4. Case Studies

**Figure 1:**
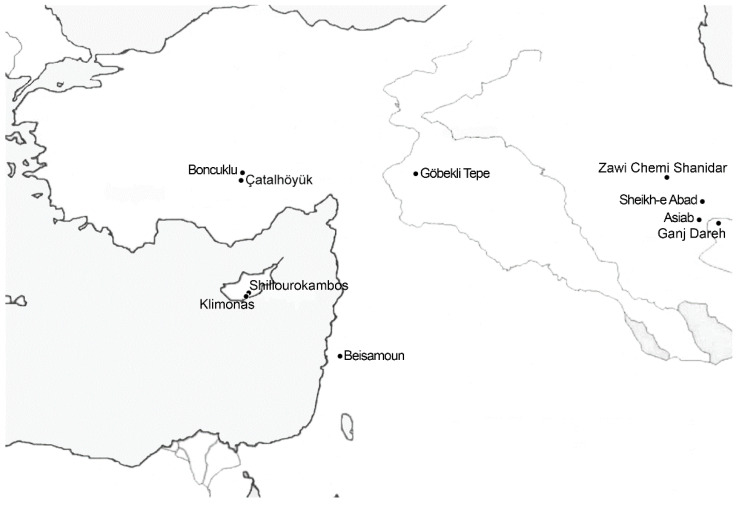
Near East with sites mentioned in the text.

Assessing prehistoric concepts such as wildness and domestication is challenging, and requires rich contextual information as well as thorough zooarchaeological analyses [51]. I will therefore focus on a few case studies where circumstances reveal decisions made by ancient people that illuminate their thinking about animal categories (Figure 1).

### 4.1. Cyprus

Cyprus presents a fascinating window into Neolithic human–animal relations. Colonized by humans in the early Holocene, the terrestrial island fauna was extremely depauperate, so these Neolithic sailors ferried over animals to populate the island. We do not know much about the boats used, but it must have been challenging to bring live animals, especially wild ones [52]. Modeling shows that it would take more than a single breeding pair to establish a species, so this was a concerted effort [53]. Most importantly, aside from likely stowaways such as mice and shrews, these animals were necessarily brought deliberately and so must have been considered essential.

During the Pleistocene, Cyprus and other Mediterranean islands possessed endemic faunas that had developed in isolation from humans and other terrestrial predators. On Cyprus, this included pygmy elephants and hippopotami. As on the other islands, these became extinct by the end of the Pleistocene. There is debate whether the first human visitors in the Epipaleolithic, right at the Pleistocene/Holocene boundary (ca. 10,000 calBC) encountered the pygmy hippopotami and perhaps hastened their demise, although recent research suggests they were already extinct [54,55,56]. If they were extinct, there would have been virtually no terrestrial mammalian animals. These first visitors appear to have stocked the island with wild boar, much as later sailors have done on many of the world’s remote islands to provide a meat supply for future journeys, e.g., [57]. While poor in native terrestrial mammals, Cyprus has rich marine resources and abundant bird life, especially during migration when it is an important stopover for birds crossing the Mediterranean. These resources are probably what drew subsequent visitors through the PPNA, who also hunted the wild boar. These naturalized boars were then locally domesticated in the early PPNB [58].

Actual settlement, as opposed to brief visits, occurred in the later PPNA and accelerated at the beginning of the PPNB, notably at Shillourokambos [59,60,61]. The late PPNA settlers at Klimonas relied heavily on the previously introduced boar and seem to have brought only domestic dogs and morphologically wild but commensal cats [61]. By the early PPNB at Shillourokambos and contemporary sites, though, the inhabitants embarked on a much more ambitious program of animal introductions. These animals include some we tend to think of as domestic (cattle, sheep, goats) and some we think of as wild (fallow deer, fox); they also started to raise pigs rather than hunt them. At this point we see only the murky beginnings of herding on the mainland, and all the animals transported were morphologically wild [62]. As a further twist, the domestic dogs introduced in the PPNA survive only as a feral population in the early PPNB, occasionally hunted and eaten [63].

On the one hand, this blurs the line between wild and domestic, for these proto-Cypriots apparently captured and transported wild animals in some quantities—a form of wildlife management that verges on herding. On the other hand, once they got to Cyprus, they appear to have herded some species (the ones we would expect, although pigs/boar, the first animals transported, were apparently hunted at first), while they set others loose to establish wild populations that they then hunted, judging from the mortality profiles.

Apparently the wild and domestic species played distinct but culturally necessary roles. At Shillourokambos, butchering and cooking traces show the foxes were skinned and then eaten [63]. Fox hides may have been required for costumes in certain ceremonies, see [64], or perhaps living foxes were essential wild companions, playing a trickster role similar to the coyote in the American West—fox remains are surprisingly common at Near Eastern Epipaleolithic and Pre-Pottery Neolithic sites [65,66,67,68]. Foxes or fox parts appear in human burials in these same periods [69,70,71], and they are frequently depicted in PPNA Upper Mesopotamia [72,73,74].

Boar and especially deer hunting may have been a crucial way of performing masculinity or demonstrating maturity. Jean-Denis Vigne and Jean Guilaine [63] suggest that a perceived distinction between wild and domestic was not established until the late PPNB, but I would argue that the contrasting treatment of fallow deer as opposed to cattle, sheep, and goats indicates an earlier distinction.

### 4.2. PPNA Upper Mesopotamia

A striking feature of the PPN throughout the Near East, and particularly in Upper Mesopotamia, is that during the time that people were committing to cultivation and herding, the imagery they produced, much of it in what might be considered sacred contexts, is not of fertility and pastoral scenes but rather wild and often dangerous animals [72,75,76,77,78]. The carvings at PPNA Göbekli Tepe are particularly spectacular and well-known, but similar imagery occurs in different media elsewhere [73,79,80]. Animals were depicted in earlier periods, but with much less emphasis on threatening teeth and claws, or venomous creatures such as snakes, scorpions, and spiders.

At this point, plant cultivation was well established in this area [81], and early experiments with herding seem likely, since it is evident in the zooarchaeological record in the early PPNB [41], confirmed by the evidence from Cyprus. I will not explore here the reasons that Neolithic people responded to the beginnings of domestication with a celebration of the wild, but simply note that bringing plants and animals into the human sphere created wildness [15]. Prior to domestication, humans were interested in other species and interacted with them, but they would not form a single category of the Wild. I suggest that this category was constructed, with associations of danger and death, in response to the development of a multispecies domestic sphere.

### 4.3. Çatalhöyük

Çatalhöyük is a large tell site on the Konya Plain in central Anatolia; the Neolithic East Mound was continuously occupied from 7100–6000 calBC. First excavated in the 1960s by James Mellaart [82,83,84,85,86], Ian Hodder directed renewed work there from 1993–2017 [87,88,89,90,91,92,93,94,95,96,97,98,99,100,101]. This discussion is based on results excavated by the Hodder project through 2008. The mound is composed of layers of closely-packed mudbrick houses. In contrast to Upper Mesopotamia, there are no public buildings; ritual as well as mundane activities occurred in and around the houses, and likely on the perimeter of the site.

Domestic sheep, goats, cattle, and pigs were available to the east when the settlement was founded but were adopted selectively. Dogs, sheep, and goats were mainly domestic from the earliest levels; the remaining species (principally cattle, equids, and a few boar and deer) are all wild, except that some domestic cattle appear in the later levels of the site [102,103,104].

Corresponding wild and domestic forms are treated differently in every case. The fairly scant wolf remains at the site are almost entirely skull (in fact mandibles and teeth) and feet, suggesting that wolves mainly came to the site as skins. The only remains from elsewhere in the body are all from the forelimb (although they are spread through the sequence): four ulnae and a humerus. One ulna is worked into a heavy point, while another had been set into an interior wall and used as a hook, perhaps for a demolished installation such as a bucranium. Two articulated paws occur in special deposits associated with rebuilding a series of superimposed houses; the adjoining midden deposits also have a high proportion of the site’s wolf remains. This kind of limited representation can indicate a taboo, where wolves could be killed but not eaten, and selected body parts were brought back for special purposes [105,106].

Dogs, on the other hand, are relatively common and remains come from throughout the body. They were occasionally eaten but usually not. Their presence is also attested by gnawed and digested bones, although the distribution of gnawed bones shows that dogs were not allowed in the houses [107]. Dogs were not buried but rather disposed of in middens or abandoned houses. Possibly those placed in houses after their use were meant to protect the inhabitants (people were buried below house floors, and in one case a puppy was placed on top of a platform with human burials below) or the house built above. The only worked dog bones are a set of 25 tooth beads strung along with one badger tooth bead and several stone beads on an anklet worn by a ten-year-old child buried in Building 44, one of the houses with a wolf paw deposit [108,109]. Thus, dogs were restricted in their movements, and perhaps subject to a different kind of taboo that prohibited eating them under ordinary circumstances but gave less power to their remains. However, they may have been valued as guardians in both life and death [110].

Domestic goats and especially sheep provided most of the meat for ordinary meals throughout the sequence. In the later periods, they are also found in feasting remains, especially those associated with the closing of houses, whereas earlier feasts mainly featured wild cattle and equids [111]. Horns or frontlets of male wild goats and sheep were sometimes built into house features, such as the wild rams in an earlier phase of a bench in Building 52 [112], later fitted with wild cattle horns; or those placed with care in abandoned houses. Horns of domestic sheep and goats sometimes occur in the deposits of feasting remains, but otherwise do not get special treatment [102,103,104,113]. The astragali of domestic sheep and goats, and to a much lesser extent of other ruminants including wild sheep and goats, are collected in the later periods into caches, some of them worked to varying degrees, for use as knucklebones in child’s play, gambling, or divination.

The only real animal burial (as opposed to parts of animals in human burials or disposal of dog remains) is a female lamb buried with an adult male human [114]. There is, however, a sense of ambivalence about the inclusion of the lamb. It was separated from the man by a mat that ran under the lamb and over the man, so that they did not actually touch. Someone held the lamb’s feet straight up while the grave was filled, presumably to keep them from falling across the man. In the Near Eastern Neolithic in general, burials of complete animals are usually domestic and often young [114]. Although the mourners seem hesitant about whether the lamb belonged in the grave, and treated it as though it posed a danger of contamination, it probably was a personal possession like other grave goods at the site, marking the lamb as property. In sum, domestic sheep and goats were used in a number of ways, but do not seem to carry the kind of spiritual power that adheres to their wild counterparts.

Wild cattle, or aurochsen, and especially wild bulls, carried strong symbolic value at Çatalhöyük, although they were always a minority of the assemblage [75,115,116,117,118,119,120,121,122,123]. Indeed, it is interesting that they are not more frequent, since their very large size indicates that the Konya Plain provided prime habitat [103]. The early Holocene Konya Plain was a mosaic of steppe and wetlands, with the aurochsen probably primarily inhabiting the steppe areas [124]. The aurochs habitat was close enough to Çatalhöyük that hunters brought all body parts back to the settlement. While their larger size means that aurochsen made a substantial contribution to the diet, they were not treated as a staple meat source. The prestige of the hunt and the social benefits of sharing out the meat may have been more important than the calories.

Cattle are a key component of feasting deposits, with males over-represented in these and other special contexts [103]. Cattle parts are frequently found in what I have termed commemorative deposits: idiosyncratic selections of items buried beneath house floors that appear to represent specific events [125]. These commemorative deposits were buried on the south and west sides of houses, while human burials are generally on the north and east, implying a certain complementarity. If humans were buried in houses so that ancestors, in the broad sense, could protect and benefit the inhabitants, it implies that these material talismans of ceremonies also had protective and beneficial power, and perhaps even that cattle were regarded as a kind of ancestor, possibly invoking a mythic past when humans and cattle were not distinct [118].

Wild bull horns were incorporated into houses in both visible and invisible ways [121]. As well as the hidden commemorative deposits, horns were sometimes concealed in walls. They were also set into benches, on pillars, and in clay heads. These displays likely served as trophies that attested to past hunts and feasts, but given the concealed horn deposits, they probably also enhanced the power of the house [122]. The only domestic cattle horn treated in a similar fashion is a single bull horn set into an internal wall, without a clay head. This is a different treatment from the other displayed horns, which are always frontlets. It was likely a hook for another installation or item, and in fact is in the same house as the wolf ulna hook.

Clearly aurochsen played a major role in the social and religious lives of the inhabitants of Çatalhöyük, and to a lesser degree of the Neolithic Near East in general. Other sites also have aurochs heads and horns placed in buildings, as well as cattle imagery, but not in the quantities we see at Çatalhöyük. Jacques Cauvin [126] argued, largely reading back from Çatalhöyük, that a bull god entered the pantheon in the PPNB of the Near East, joining the goddess who had emerged earlier. I am more inclined to see bulls as powerful spirit beings than gods, but in any case, most agree that they had religious significance.

The founders of Çatalhöyük could have brought domestic cattle as well as sheep and goats, or captured and herded local aurochsen, but they did not. Benjamin Arbuckle’s recent meta-analysis of faunal data from Neolithic Anatolia shows that, in fact, domestic cattle skipped over Central Anatolia (not only Çatalhöyük) and were herded to the west for about 500 years before they were adopted in Central Anatolia [127,128,129,130]. They reached the Lake District to the west of Çatalhöyük by 7000 calBC, only shortly after Çatalhöyük was settled. Thus, the lack of domestic cattle at Çatalhöyük and other Central Anatolian sites was a deliberate choice, a refusal to treat cattle like domestic sheep and goats. I argue that the intensity and importance of the relationship of humans with wild cattle persons, integral to the protection and prosperity of house and household, perhaps felt as kin, was so great and so spiritually central that it was unthinkable to render other cattle into property, or to care for them like perpetual children [118]. At Çatalhöyük, the aurochsen cared for the people. The distinction between wild and domestic cattle was stark for them, and the relations of herding were offensive in a way that was not true for sheep and goats.

However, eventually Central Anatolians, including the residents of Çatalhöyük, did accept domestic cattle. At Çatalhöyük, this happens at a time of many changes at the site [131,132]. These include more independence of houses in production, and more marking of individuals seen, for instance, in a proliferation of types and amounts of adornment, and in the movement of motifs from large objects built into houses (reliefs, bucrania) to small portable objects (stamp seals, pottery decoration). Feasting intensifies and diversifies, and may become more competitive [111]. Perhaps domestic cattle were adopted to supplement aurochsen in these feasts, and sheep and goats were also included. Bone tool types in the earlier levels were shared with sites to the east, while in the later levels they are shared with sites to the west [133]. Harvey Whitehouse and Ian Hodder [134] argue that religious practice shifted from infrequent, dramatic ceremonies in the early levels to frequent, less dramatic rituals in the later levels. These religious and ideological changes apparently reduced the spiritual power of aurochsen, although they did not eliminate it as cattle remains still appear in special deposits and in fact some of the most dramatic cattle depictions come from the later levels [123].

**Figure 2:**
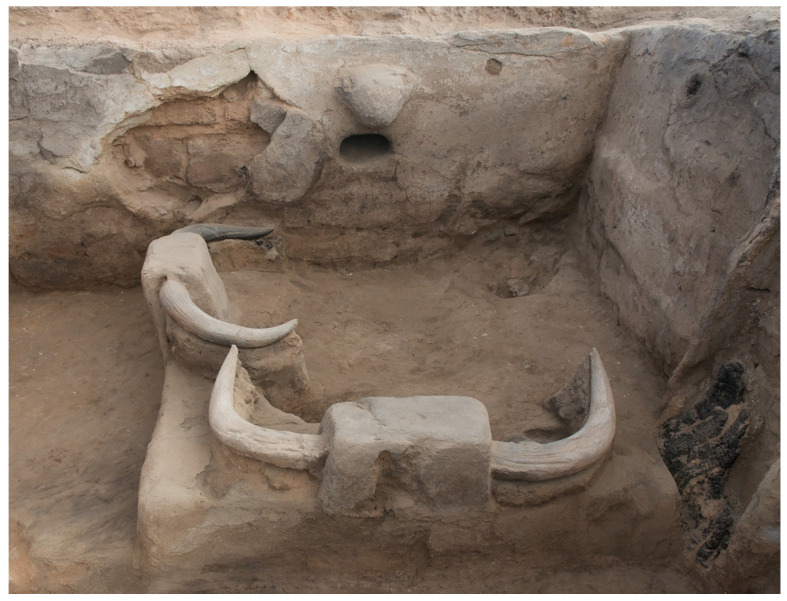
Aurochs horns on pillars on northeast platform in burnt Building 77 (photo by Jason Quinlan, Çatalhöyük Research Project).

These changes were contested. The inception of these changes and the appearance of the first domestic cattle were marked by an intensification of wild cattle displays, as if to assert the power of the aurochs against the challenge of cattle herding [104]. Where previously special deposits might contain one or two horns or scapulae, now they often occur in much larger quantities (as many as eight scapulae or 22 horns). The horns set in benches and pillars are largely a feature of these transitional levels as well (Figure 2). Many houses were burned at the end of their life cycle at this time, and not before or after. The burning appears to be a deliberate and dramatic way of closing the houses rather than accidental fires or the result of warfare [131,135,136]. As noted, when domestic cattle were adopted they were used in feasts but not in displays (with the possible exception of the single horn set in the wall of Building 1). Moreover, the addition of cattle herding did not increase the numbers of cattle at the site. Rather, the total number of cattle, wild and domestic, remained about the same, while there was a sharp increase in domestic sheep [104].

Arbuckle’s analysis also showed that domestic pigs similarly skipped over Central Anatolia to spread to the Lake District at the same time as domestic cattle, although they were a bit slower than cattle in reaching northwest Anatolia [127,129]. They were also much slower to be adopted in Central Anatolia, where they do not appear until the Chalcolithic (ca. 4500 calBC). I suggest there was a different dynamic at work here, but again there was a distinction in the treatment of wild and domestic forms. Wild boar were less common than one would expect at Çatalhöyük, given that the marshy habitat surrounding the site in the early Holocene should have favored them. They were much more prevalent at the earlier Boncuklu site 10 km away [137]. Wild boar remains from throughout the body were found in areas on the edge of the Çatalhöyük settlement, and boar were somewhat commoner there. On site, they are heavily biased to heads. This pattern suggests a partial taboo, where some categories of people could kill and eat boar, but they did so off-site or on the periphery, bringing only select pieces into the settlement to prevent contact with categories of people to whom boar were taboo [105,106].

Boar remains, mainly from the head and primarily mandibles, are found in special deposits and set into architecture, but in much smaller quantities than cattle [123]. Boar are depicted at Çatalhöyük and elsewhere [78,123]. These treatments, as well as the taboo, mark them as spiritually powerful animals, but they do not seem to have held the same kind of kinship relation to humans that cattle did. Domestic pigs may have been similar enough to invoke the taboo but lacked the symbolic cachet of wild boar.

### 4.4. Elsewhere in the Fertile Crescent

I have focused on three relatively restricted case studies that illustrate, in differing ways, conceptual distinctions between wild and domestic animals. Finds elsewhere in the greater Fertile Crescent region also attest to these distinctions.

In the Levant, aurochsen were often the featured food at feasts [138,139]. Aurochs remains, especially skulls and horns, were placed in buildings as foundation, closing, or other special deposits, or were arranged in pits [140,141,142]. At PPNC Beisamoun, a wild boar skull was placed in a human grave, and an intact wild boar was apparently buried on its own [140]. It appears that as domestic livestock began to be herded, aurochsen played less of a role in feasting while the body parts of these and other wild animals were more often deployed in special deposits [138,139].

At the other end of the Fertile Crescent in the Zagros region, aurochsen seem to carry less spiritual power, with wild boar and caprines along with large raptors dominating special deposits. At Asiab, at least 19 wild boar skulls, a bear skull, and red deer antler were carefully arranged in a pit [143]. At Sheik-e Abad, wild goat and wild sheep skulls were likewise arranged along with a large bird bone on a room floor [144,145]. The combination of wild goats and raptors recurs at Zawi Chemi Shanidar [146,147], while at Ganj Dareh two sheep skulls were mounted vertically on a wall [148,149].

## 5. Conclusions

In this brief review of early domesticates and their wild counterparts in the Neolithic Near East, it is apparent that while the roles of both wild and domestic animals vary by taxon (and in fact by sex in at least some cases, and no doubt in other ways), in every case wild and domestic forms are treated and surely regarded differently. Indeed, the early settlers of Cyprus distinguished between wild and domestic animals even as they secured supplies of both to populate the island. As well as establishing their herds, they needed some animals to be there, but to be wild. On the other hand, the nature of human relations with specific wild animals impeded the adoption of some domesticates in central Anatolia and may have shaped the spread of herding elsewhere. Regional variation in which wild species held special spiritual power or symbolic cachet are also apparent. Indeed, there are local differences at a finer geographic scale and even from site to site, as well as through time, that are too complex to follow here but surely reflect belief and ritual practice in each community.

Domestic animals were useful but received very little attention in the Neolithic symbolic sphere; the same was true of plant agriculture even as it created profound social changes. Perhaps in active resistance to the new practices of cultivation and herding, or at any rate in contrast to them, the people of the earlier Neolithic instead intensified their ritual relations with wild animals. Hunting may have become more performative as it became less essential to subsistence. Domestication created not only new kinds of relationships with animals and the land, but also a new category of the Wild, set off from the Domestic of field and herd as well as settlement. The line between nature and culture was drawn more strongly.

## Data Availability

Not applicable.

## References

[1] Alberti B. (2016). Archaeologies of ontology. Ann. Rev. Anthro..

[2] Conty A.F. (2021). Animism in the Anthropocene. Theory Cult. Soc..

[3] Harris O.J.T., Cipolla C.N. (2017). Archaeological Theory in the New Millennium: Introducing Current Perspectives.

[4] Kohn E.O. (2015). Anthropology of ontologies. Ann. Rev. Anthro..

[5] Watts C. (2013). Relational Archaeologies: Humans, Animals, Things.

[6] Boyd B. (2017). Archaeology and human–animal relations: Thinking through anthropocentrism. Ann. Rev. Anthro..

[7] Descola P. (2005). Par-Delà Nature et Culture.

[8] O’Connor T.P. (1997). Working at relationships: Another look at animal domestication. Antiquity.

[9] Bökönyi S., Ucko P.J., Dimbleby G.W. (1969). Archaeological problems and methods of recognizing animal domestication. The Domestication and Exploitation of Plants and Animals.

[10] Clutton-Brock J. (1992). The process of domestication. Mamm. Rev..

[11] Ducos P., Meadow R.H., Zeder M.A. (1978). “Domestication” defined and methodological approaches to its recognition in faunal assemblages. Approaches to Faunal Analysis in the Middle East.

[12] Jarman M.R., Hutchinson J., Clark J.G.D., Jope E.M., Riley R. (1977). Early animal husbandry. The Early History of Agriculture.

[13] Losey R.J. (2022). Domestication is not an ancient moment of selection for prosociality: Insights from dogs and modern humans. J. Soc. Arch..

[14] Armstrong Oma K., Böhm A., Ullrich J. (2019). First encounters: Domestication as steps of becoming. Animal Encounters.

[15] Russell N. (2002). The wild side of animal domestication. Soc. Anim..

[16] Zeder M.A. (2015). Core questions in domestication research. Proc. Natl. Acad. Sci. USA.

[17] Clark N., Cassidy R., Mullin M.H. (2007). Animal interface: The generosity of domestication. Where the Wild Things Are Now: Domestication Reconsidered.

[18] Armstrong Oma K. (2010). Between trust and domination: Social contracts between humans and animals. World Archaeol..

[19] Tani Y. (2017). God, Man and Domesticated Animals: The Birth of Shepherds and their Descendants in the Ancient Near East.

[20] Mlekuž D. (2013). The birth of the herd. Soc. Anim..

[21] Trut L.N., Oskina I.N., Kharlamova A.V. (2009). Animal evolution during domestication: The domesticated fox as a model. BioEssays.

[22] Clutton-Brock J., Manning A., Serpell J.A. (1994). The unnatural world: Behavioural aspects of humans and animals in the process of domestication. Animals and Human Society: Changing Perspectives.

[23] Tchernov E., Horwitz L.R.K. (1991). Body size diminution under domestication: Unconscious selection in primeval domesticates. J. Anthropol. Archaeol..

[24] Hemmer H. (1990). Domestication: The Decline of Environmental Appreciation.

[25] Zeder M.A. (2012). The domestication of animals. J. Anthro. Res..

[26] Willerslev R. (2007). Soul Hunters: Hunting, Animism, and Personhood among the Siberian Yukaghirs.

[27] Brightman R.A. (1993). Grateful Prey: Rock Cree Human-Animal Relationships.

[28] Bird-David N.H., Milton K. (1993). Tribal metaphorization of human-nature relatedness: A comparative analysis. Environmentalism: The View from Anthropology.

[29] Guenther M.J. (2020). Human-Animal Relationships in San and Hunter-Gatherer Cosmology.

[30] Guenther M.J. (2020). Human-Animal Relationships in San and Hunter-Gatherer Cosmology.

[31] Brown L.A., Emery K.F. (2008). Negotiations with the animate forest: Hunting shrines in the Guatemalan highlands. J. Archaeol. Method Theory.

[32] McNiven I.J., Watts C. (2013). Between the living and the dead: Relational ontologies and the ritual dimensions of dugong hunting across Torres Strait. Relational Archaeologies: Humans, Animals, Things.

[33] Anderson V.D. (2004). Creatures of Empire: How Domestic Animals Transformed Early America.

[34] Naveh D., Bird-David N.H. (2014). How persons become things: Economic and epistemological changes among Nayaka hunter-gatherers. J. Roy. Anth. Inst..

[35] Ingold T., Manning A., Serpell J.A. (1994). From trust to domination: An alternative history of human-animal relations. Animals and Human Society: Changing Perspectives.

[36] Kent S., Kent S. (1989). Cross-cultural perceptions of farmers as hunters and the value of meat. Farmers as Hunters: The Implications of Sedentism.

[37] Jennbert K., Andersson Cederholm E., Björck A., Jennbert K., Lönngren A.-S. (2014). Certain humans, certain animals: Attitudes in the long term. Exploring the Animal Turn: Human-Animal Relations in Science, Society and Culture.

[38] Tambiah S.J. (1969). Animals are good to think and good to prohibit. Ethnology.

[39] Brotherston G., Clutton-Brock J., Ucko P.J. (1989). Andean pastoralism and Inca ideology. The Walking Larder: Patterns of Domestication, Pastoralism, and Predation.

[40] Rosman A., Rubel P.G., Kent S. (1989). Stalking the wild pig: Hunting and horticulture in Papua New Guinea. Farmers as Hunters: The Implications of Sedentism.

[41] Arbuckle B.S. (2018). Early history of animal domestication in southwest Asia. Oxford Research Encyclopedia of Environmental Science.

[42] Hongo H., Meadow R.H., Öksüz B., İlgezdi G., Buitenhuis H., Choyke A.M., Martin L., Bartosiewicz L., Mashkour M. (2005). Sheep and goat remains from Çayönü Tepesi, southeastern Anatolia. Archaeozoology of the Near East VI: Proceedings of the Sixth International Symposium on the Archaeozoology of Southwestern Asia and Adjacent Areas.

[43] Grupe G., Peters J., Vila E., Gourichon L., Choyke A.M., Buitenhuis H. (2008). Feeding humans and animals at Pre-Pottery Neolithic Nevalı Çori (SE-Anatolia) as evidenced by stable isotope analysis. Archaeozoology of the Near East VIII.

[44] Helmer D., Vila E., Gourichon L., Choyke A.M., Buitenhuis H. (2008). Révision de la faune de Cafer Höyük (Malatya, Turquie): Apports des méthodes de l’analyse des mélanges et de l’analyse de Kernel à la mise en évidence de la domestication. Archaeozoology of the Near East VIII.

[45] Zeder M.A., Hesse B.C. (2000). The initial domestication of goats (*Capra hircus*) in the Zagros Mountains 10,000 years ago. Science.

[46] Helmer D., Gourichon L., Monchot H., Peters J., Saña Seguí M., Vigne J.-D., Peters J., Helmer D. (2005). Identifying early domestic cattle from Pre-Pottery Neolithic sites on the Middle Euphrates using sexual dimorphism. The First Steps of Animal Domestication.

[47] Saña Seguí M., Tornero C., Molist Montaña M. (2013). La gestión de los recursos animales en Tell Halula: Nuevas hipótesis y aproximaciones metodológicas para el estudio de domesticación animal y producción ganadera. Tell Halula: Un Poblado de los Primeros Agricultores en el Valle del Éufrates, Siria.

[48] Ervynck A., Dobney K.M., Hongo H., Meadow R.H. (2001). Born free? New evidence for the status of *Sus scrofa* at Neolithic Çayönü Tepesi (southeastern Anatolia, Turkey). Paléorient.

[49] Lösch S., Grupe G., Peters J. (2006). Stable isotopes and dietary adaptations in humans and animals at pre-pottery Neolithic Nevalı Çori, southeast Anatolia. Am. J. Phys. Anthropol..

[50] Peters J., von den Dreisch A., Helmer D., Vigne J.-D., Peters J., Helmer D. (2005). The upper Euphrates-Tigris basin: Cradle of agro-pastoralism?. The First Steps of Animal Domestication.

[51] Russell N. (2012). Social Zooarchaeology: Humans and Animals in Prehistory.

[52] Vigne J.-D. (2014). The origins of mammals on the Mediterranean islands as an indicator of early voyaging. Eurasian Prehistory.

[53] Ducos P., Mashkour M., Choyke A.M., Buitenhuis H., Poplin F. (2000). The introduction of animals by man in Cyprus: An alternative to the Noah’s Ark model. Archaeozoology of the Near East IVA: Proceedings of the Fourth International Symposium on the Archaeozoology of Southwestern Asia and Adjacent Areas.

[54] Simmons A.H., Phoca-Cosmetatou N. (2011). Re-writing the colonisation of Cyprus: Tales of hippo hunters and cow herders. The First Mediterranean Islanders: Initial Occupation and Survival Strategies.

[55] Vigne J.-D., Colledge S.M., Conolly J., Dobney K.M., Manning K., Shennan S.J. (2013). Domestication process and domestic ungulates: New observations from Cyprus. The Origins and Spread of Domestic Animals in Southwest Asia and Europe.

[56] Zazzo A., Lebon M., Quiles A., Reiche I., Vigne J.-D. (2015). Direct dating and physico-chemical analyses cast doubts on the coexistence of humans and dwarf hippos in Cyprus. PLoS ONE.

[57] Ruiz-Ballesteros E., del Campo Tejedor A. (2022). Eradicate to construct nature. Goats, rats, and humans in Floreana (Galapagos Islands). Ethnos.

[58] Cucchi T., Domont A., Harbers H., Evin A., Alcàntara Fors R., Saña Seguí M., Leduc C., Guidez A., Bridault A., Hongo H. (2021). Bones geometric morphometrics illustrate 10th millennium cal. BP domestication of autochthonous Cypriot wild boar (*Sus scrofa circeus* nov. ssp). Sci. Rep..

[59] Vigne J.-D., Briois F., Zazzo A., Willcox G.H., Cucchi T., Thiébault S., Carrère I., Franel Y., Touquet R., Martin C. (2012). First wave of cultivators spread to Cyprus at least 10,600 y ago. Proc. Natl. Acad. Sci. USA.

[60] Guilaine J., Briois F., Vigne J.-D. (2011). Shillourokambos: Un Établissement Néolithique Pré-céramique à Chypre. Les Fouilles du Secteur 1.

[61] Vigne J.-D., Briois F., Cucchi T., Franel Y., Mylona P., Tengberg M., Touquet R., Wattez J., Willcox G.H., Zazzo A., Vigne J.-D., Briois F., Tengberg M. (2017). Klimonas, a late PPNA hunter-cultivator village in Cyprus: New results. Nouvelles Données sur les Débuts du Néolithique à Chypre.

[62] Vigne J.-D., Carrère I., Briois F., Guilaine J. (2011). The early process of mammal domestication in the Near East: New evidence from the Pre-Neolithic and Pre-Pottery Neolithic in Cyprus. Curr. Anthropol..

[63] Vigne J.-D., Guilaine J. (2004). Les premiers animaux de compagnie, 8500 ans avant notre ère? … ou comment j’ai mangé mon chat, mon chien et mon renard. Anthropozoologica.

[64] Collins P.W. (1991). Interaction between island foxes (*Urocyon littoralis*) and Native Americans on islands off the coast of southern California: II. Ethnographic, archaeological, and historic evidence. J. Ethnobiol..

[65] Carruthers D., Buitenhuis H., Choyke A.M., Mashkour M., Al-Shiyab A.H. (2002). The Dana-Faynan-Ghuwayr Early Prehistory Project: Preliminary animal bone report on mammals from Wadi Faynan 16. Archaeozoology of the Near East V: Proceedings of the Fifth International Symposium on the Archaeozoology of Southwestern Asia and Adjacent Areas.

[66] Hesse B.C., Clutton-Brock J., Grigson C. (1984). These are our goats: The origins of herding in west central Iran. Animals and Archaeology: 3. Early Herders and their Flocks.

[67] Horwitz L.R.K., Tchernov E., Ducos P., Becker C., Von Den Driesch A., Martin L., Garrard A.N. (1999). Animal domestication in the southern Levant. Paléorient.

[68] Yeshurun R., Bar-Oz G., Weinstein-Evron M. (2009). The role of foxes in the Natufian economy: A view from Mount Carmel, Israel. Before Farming.

[69] Reshef H., Anton M., Bocquentin F., Vardi J., Khalaily H., Davis L., Bar-Oz G., Marom N. (2019). Tails of animism: A joint burial of humans and foxes in Pre-Pottery Neolithic Motza, Israel. Antiquity.

[70] Maher L.A., Stock J.T., Finney S., Heywood J.J.N., Miracle P.T., Banning E.B. (2011). A unique human-fox burial from a pre-Natufian cemetery in the Levant (Jordan). PLoS ONE.

[71] Horwitz L.R.K., Goring-Morris A.N. (2004). Animals and ritual during the Levantine PPNB: A case study from the site of Kfar Hahoresh, Israel. Anthropozoologica.

[72] Gourichon L., Helmer D., Peters J., Sidéra I., Vila E., Erikson P. (2006). À la croisée des pratiques cynégétiques et de l’iconographie des animaux sauvages: Haute et moyen Euphrate–Xe et IXe millénaires av. J.-C. La Chasse: Pratiques Sociales et Symboliques.

[73] Benz M., Bauer J. (2015). On scorpions, birds, and snakes—Evidence for shamanism in Northern Mesopotamia during the Early Holocene. J. Ritual Stud..

[74] Peters J., Schmidt K. (2004). Animals in the symbolic world of Pre-Pottery Neolithic Göbekli Tepe, south-eastern Turkey: A preliminary assessment. Anthropozoologica.

[75] Hodder I., Meskell L. (2011). A “curious and sometimes a trifle macabre artistry”. Curr. Anthropol..

[76] Borić D., Watts C. (2013). Theater of predation: Beneath the skin of Göbekli Tepe images. Relational Archaeologies: Humans, Animals, Things.

[77] Fagan A. (2017). Hungry architecture: Spaces of consumption and predation at Göbekli Tepe. World Archaeol..

[78] Helmer D., Gourichon L., Stordeur D. (2004). À l’aube de la domestication animale: Imaginaire et symbolisme animal dans les premières sociétés néolithiques du nord du Proche-Orient. Anthropozoologica.

[79] Schmidt K. (2012). Göbekli Tepe: A Stone Age Sanctuary in South-Eastern Anatolia.

[80] Stordeur D., Guilaine J. (2003). Symboles et imaginaire des premières cultures néolithiques du Proche-Orient (haute et moyenne vallée de l’Euphrate). Arts et Symboles du Néolithique à la Protohistoire.

[81] Kabukcu C., Asouti E., Pöllath N., Peters J., Karul N. (2021). Pathways to plant domestication in Southeast Anatolia based on new data from aceramic Neolithic Gusir Höyük. Sci. Rep..

[82] Mellaart J. (1962). Excavations at Çatal Hüyük, 1961: First preliminary report. Anatol. Stud..

[83] Mellaart J. (1963). Excavations at Çatal Hüyük, 1962: Second preliminary report. Anatol. Stud..

[84] Mellaart J. (1964). Excavations at Çatal Hüyük, 1963: Third preliminary report. Anatol. Stud..

[85] Mellaart J. (1966). Excavations at Çatal Hüyük, 1965: Fourth preliminary report. Anatol. Stud..

[86] Mellaart J. (1967). Çatal Hüyük: A Neolithic Town in Anatolia.

[87] Hodder I. (1996). On the Surface: Çatalhöyük 1993–95.

[88] Hodder I. (2005). Changing Materialities at Çatalhöyük: Reports from the 1995–99 Seasons.

[89] Hodder I. (2005). Inhabiting Çatalhöyük: Reports from the 1995–99 Seasons.

[90] Hodder I. (2006). Çatalhöyük Perspectives: Themes from the 1995–99 Seasons.

[91] Hodder I. (2006). The Leopard’s Tale: Revealing the Mysteries of Çatalhöyük.

[92] Hodder I. (2007). Excavating Çatalhöyük: South, North and KOPAL Area Reports from the 1995–99 Seasons.

[93] Hodder I. (2013). Substantive Technologies at Çatalhöyük: Reports from the 2000–2008 Seasons.

[94] Hodder I. (2013). Humans and Landscapes of Çatalhöyük: Reports from the 2000–2008 Seasons.

[95] Hodder I. (2014). Integrating Çatalhöyük: Themes from the 2000–2008 Seasons.

[96] Hodder I. (2014). Çatalhöyük Excavations: The 2000–2008 Seasons.

[97] Hodder I. (2020). Twenty-five years of research at Çatalhöyük. Near East. Archaeol..

[98] Hodder I. (2021). Peopling the Landscape of Çatalhöyük: Reports from the 2009–2017 Seasons.

[99] Hodder I. (2021). The Matter of Çatalhöyük: Reports from the 2009–2017 Seasons.

[100] Hodder I., Tsoraki C. (2021). Communities at Work: The Making of Çatalhöyük.

[101] Hodder I. (2022). Çatalhöyük Excavations: The 2009–2017 Seasons.

[102] Russell N., Tringham R.E., Stevanović M. (2012). Mammals from the BACH Area. Last House on the Hill: BACH Area Reports from Çatalhöyük, Turkey.

[103] Russell N., Martin L., Hodder I. (2005). The Çatalhöyük mammal remains. Inhabiting Çatalhöyük: Reports from the 1995–1999 Seasons.

[104] Russell N., Twiss K.C., Orton D.C., Demirergi G.A., Hodder I. (2013). More on the Çatalhöyük mammal remains. Humans and Landscapes of Çatalhöyük: Reports from the 2000–2008 Seasons.

[105] Politis G.G., Saunders N.J. (2002). Archaeological correlates of ideological activity: Food taboos and spirit-animals in an Amazonian hunter-gatherer society. Consuming Passions and Patterns of Consumption, Miracle, P.T., Milner, N., Eds..

[106] Russell N., Ivanova M., Athanassov B., Petrova V., Takorova D., Stockhammer P.W. (2018). Neolithic taboos in Anatolia and Southeast Europe. Social Dimensions of Food in the Prehistoric Balkans.

[107] Russell N., Twiss K.C., Mashkour M., Beech M. (2017). Digesting the data: Dogs as taphonomic agents at Neolithic Çatalhöyük, Turkey. Archaeozoology of the Near East 9.

[108] Russell N., Griffitts J.L., Hodder I. (2013). Çatalhöyük worked bone: South and 4040 Areas. Substantive Technologies at Çatalhöyük: Reports from the 2000–2008 Seasons.

[109] Bains R., Vasić M., Bar-Yosef D.E., Russell N., Wright K.I., Doherty C., Hodder I. (2013). A technological approach to the study of personal ornamentation and social expression at Çatalhöyük. Substantive Technologies at Çatalhöyük: Reports from the 2000–2008 Seasons.

[110] Russell N., Bethke B., Burtt A. (2020). Scavenger and sentry: The roles of dogs at Çatalhöyük, Turkey, in the context of the Near Eastern Neolithic. Dogs: Archaeology beyond Domestication.

[111] Russell N., Twiss K.C., Orton D.C., Demirergi G.A., de Cupere B., Linseele V., Hamilton-Dyer S. (2014). Changing animal use at Neolithic Çatalhöyük, Turkey. Archaeozoology of the Near East X.

[112] Tung B. Excavations in the North Area, 2013; Çatalhöyük Research Project, Archive Report 2013. https://www.catalhoyuk.com/sites/default/files/media/pdf/Archive_Report_2013.pdf.

[113] Russell N., Wright K.I., Carter T., Ketchum S., Ryan P., Yalman E.N., Regan R., Stevanović M., Milić M., Hodder I. (2014). Bringing down the house: House closing deposits at Çatalhöyük. Integrating Çatalhöyük: Themes from the 2000–2008 Seasons.

[114] Russell N., Düring B.S. (2006). Worthy is the lamb: A double burial at Neolithic Çatalhöyük (Turkey). Paléorient.

[115] Der L. (2021). Tracing human-animal relations across time and space through animal materialisations. The Matter of Çatalhöyük: Reports from the 2009–2017 Seasons, Hodder, I., Ed..

[116] Forest J.-D. (1993). Çatal Hüyük et son décor: Pour le déchiffrement d’un code symbolique. Anatolia Antiq..

[117] Girard R., Antonello P., Gifford P. (2015). Animal scapegoating at Çatalhoÿük. How We Became Human: Mimetic Theory and the Science of Evolutionary Origins.

[118] Russell N., Wright E., Ginja C. (2022). Cattle for the ancestors at Neolithic Çatalhöyük, Turkey. Cattle and People: Interdisciplinary Approaches to an Ancient Relationship.

[119] Shults F.L., Hodder I. (2014). Excavating theogonies: Anthropomorphic promiscuity and sociographic prudery in the Neolithic and now. Religion at Work in a Neolithic Society: Vital Matters.

[120] Testart A. (2006). Interprétation symbolique et interprétation religieuse en archéologie: L’exemple du taureau à Çatal Höyük. Paléorient.

[121] Twiss K.C., Russell N. (2009). Taking the bull by the horns: Ideology, masculinity, and cattle horns at Çatalhöyük (Turkey). Paléorient.

[122] Weismantel M.J., Hodder I. (2014). The hau of the house. Religion at Work in a Neolithic Society: Vital Matters.

[123] Russell N., Meece S., Hodder I. (2006). Animal representations and animal remains at Çatalhöyük. Çatalhöyük Perspectives: Reports from the 1995–99 Seasons.

[124] Wainwright J., Ayala G. (2021). Holistic approaches to palaeohydrology: Reconstructing and modelling the Neolithic River Çarşamba and the riverscape of Çatalhöyük, Turkey. Holocene.

[125] Russell N., Martin L., Twiss K.C. (2009). Building memories: Commemorative deposits at Çatalhöyük. Anthropozoologica.

[126] Cauvin J. (1994). Naissance des Divinités, Naissance de l’Agriculture: La Révolution des Symboles au Néolithique.

[127] Arbuckle B.S. (2013). The late adoption of cattle and pig husbandry in Neolithic Central Turkey. J. Archaeol. Sci..

[128] Arbuckle B.S. (2014). Pace and process in the emergence of animal husbandry in Neolithic Southwest Asia. Bioarchaeology Near East.

[129] Arbuckle B.S., Kansa S.W., Kansa E.C., Orton D.C., Çakırlar C., Gourichon L., Atıcı A.L., Galik A., Marciniak A., Mulville J.A. (2014). Data sharing reveals complexity in the spread of domestic animals westward across Neolithic Turkey. PLoS ONE.

[130] Arbuckle B.S., Makarewicz C.A. (2009). The early management of cattle (*Bos taurus*) in Neolithic central Anatolia. Antiquity.

[131] Hodder I. (2014). Çatalhöyük: The leopard changes its spots. A summary of recent work. Anatol. Stud..

[132] Hodder I., Doherty C., Hodder I. (2014). Temporal trends: The shapes and narratives of cultural change at Çatalhöyük. Integrating Çatalhöyük: Themes from the 2000–2008 Seasons.

[133] Russell N. (2016). Anatolian Neolithic bone tools. Der Anschnitt.

[134] Whitehouse H., Hodder I., Hodder I. (2010). Modes of religiosity at Çatalhöyük. Religion in the Emergence of Civilization: Çatalhöyük as a Case Study.

[135] Harrison K. (2013). The application of forensic fire investigation techniques in the archaeological record. J. Archaeol. Sci..

[136] Twiss K.C., Bogaard A., Bogdan D., Carter T., Charles M.P., Farid S., Russell N., Stevanović M., Yalman E.N., Yeomans L. (2008). Arson or accident? The burning of a Neolithic house at Çatalhöyük, Turkey. J. Field Archaeol..

[137] Baird D., Fairbairn A.S., Jenkins E., Martin L., Middleton C., Pearson J.A., Asouti E., Edwards Y.H., Kabukcu C., Mustafaoğlu G. (2018). Agricultural origins on the Anatolian plateau. Proc. Natl. Acad. Sci. USA.

[138] Meier J.S., Goring-Morris A.N., Munro N.D. (2017). Aurochs bone deposits at Kfar HaHoresh and the southern Levant across the agricultural transition. Antiquity.

[139] Twiss K.C. (2008). Transformations in an early agricultural society: Feasting in the southern Levantine Pre-Pottery Neolithic. J. Anthropol. Archaeol..

[140] Bocquentin F., Khalaily H., Boaretto E., Dubreuil L., Schechter H.C., Bar-Yosef Mayer D.E., Greenberg H., Berna F., Anton M., Borrell F., Khalaily H., Re’em A., Vardi J., Milevski I. (2020). Between two worlds: The PPNB-PPNC transition in the central Levant as seen through discoveries at Beisamoun. The Mega Project at Motza (Moẓa): The Neolithic and Later Occupations up to the 20th Century.

[141] Kirkbride D. (1966). Five seasons at the Pre-Pottery Neolithic village of Beidha in Jordan. Palest. Explor. Q..

[142] Lechevallier M., Ronen A. (1985). Le Site Natoufien-Khiamien de Hatoula près de Latroun, Israel: Fouilles 1980–1982: Rapport Préliminaire.

[143] Bangsgaard P., Yeomans L., Darabi H., Gregersen K.M., Olsen J.V., Richter T., Mortensen P. (2019). Feasting on wild boar in the Early Neolithic. Evidence from an 11,400-year-old placed deposit at Tappeh Asiab, central Zagros. Camb. Archaeol. J..

[144] Matthews R.J., Mohammadifar Y., Matthews W., Astin T., Cole G., Darabi H., Daryaee T., Lelek Tvetmarken C., Shillito L.-M., Taylor H., Matthews R.J., Matthews W., Mohammadifar Y. (2013). Excavations, surface collection, and geophysics at Sheikh-e Abad. The Earliest Neolithic of Iran: 2008 Excavations at Sheikh-E Abad and Jani.

[145] Bendrey R., Cole G., Lelek Tvetmarken C., Matthews R.J., Matthews W., Mohammadifar Y. (2013). Zooarchaeology: Preliminary assessment of the animal bones. The Earliest Neolithic of Iran: 2008 Excavations at Sheikh-E Abad and Jani.

[146] Solecki R.L., McGovern T.H., Diamond S. (1980). Predatory birds and prehistoric man. Theory and Practice: Essays Presented to Gene Weltfish.

[147] Zeder M.A., Spitzer M.D. (2016). New insights into broad spectrum communities of the Early Holocene Near East: The birds of Hallan Çemi. Quat. Sci. Rev..

[148] Smith P.E.L., Bagherzadeh F. (1976). Reflections on four seasons of excavations at Tappeh Ganj Dareh. Proceedings of the IVth Annual Symposium on Archaeological Research in Iran: 3–8 November 1975.

[149] Smith P.E.L. (1990). Architectural innovation and experimentation at Ganj Dareh, Iran. World Archaeol..

